# Ceramide Mediates Ox-LDL-Induced Human Vascular Smooth Muscle Cell Calcification via p38 Mitogen-Activated Protein Kinase Signaling

**DOI:** 10.1371/journal.pone.0082379

**Published:** 2013-12-16

**Authors:** Lizhen Liao, Qin Zhou, Yan Song, Weikang Wu, Huimin Yu, Sheng Wang, Yanling Chen, Meihong Ye, Lihe Lu

**Affiliations:** 1 Department of Pathophysiolgy, Zhongshan Medical School, Sun Yat-Sen University, Guangzhou, China; 2 Department of Anesthesiology, The First Affiliated Hospital, Sun Yat-Sen University, Guangzhou, China; 3 Department of Cardiology, Guangdong General Hospital, Guangdong Academy of Medical Sciences and Guangdong Cardiovascular Institute, Guangzhou, China; 4 Department of Anesthesiology, Guangdong General Hospital, Guangdong Academy of Medical Sciences and Guangdong Cardiovascular Institute, Guangzhou, China; Leiden University Medical Center, The Netherlands

## Abstract

Vascular calcification is associated with significant cardiovascular morbidity and mortality, and has been demonstrated as an actively regulated process resembling bone formation. Oxidized low density lipoprotein (Ox-LDL) has been identified as a regulatory factor involved in calcification of vascular smooth muscle cells (VSMCs). Additionally, over-expression of recombinant human neutral sphingomyelinase (N-SMase) has been shown to stimulate VSMC apoptosis, which plays an important role in the progression of vascular calcification. The aim of this study is to investigate whether ceramide regulates Ox-LDL-induced calcification of VSMCs via activation of p38 mitogen-activated protein kinase (MAPK) pathway. Ox-LDL increased the activity of N-SMase and the level of ceramide in cultured VSMCs. Calcification and the osteogenic transcription factor, Msx2 mRNA expression were reduced by N-SMase inhibitor, GW4869 in the presence of Ox-LDL. Usage of GW4869 inhibited Ox-LDL-induced apoptosis in VSMCs, an effect which was reversed by C2-ceramide. Additionally, C2-ceramide treatment accelerated VSMC calcification, with a concomitant increase in ALP activity. Furthermore, C2-ceramide treatment enhanced Ox-LDL-induced VSMC calcification. Addition of caspase inhibitor, ZVAD-fmk attenuated Ox-LDL-induced calcification. Both Ox-LDL and C2-ceramide treatment increased the phosphorylation of p38 MAPK. Inhibition of p38 MAPK by SB203580 attenuated Ox-LDL-induced calcification of VSMCs. These data suggest that Ox-LDL activates N-SMase-ceramide signaling pathway, and stimulates phosphorylation of p38 MAPK, leading to apoptosis in VSMCs, which initiates VSMC calcification.

## Introduction

Vascular calcification is a prominent complication of chronic diseases including atherosclerosis, diabetes and chronic kidney disease [1]–[3]. It is an increased risk factor for the morbidity and mortality of cardiovascular disease [4], [5] and highly associated with atherosclerotic plaque stability and burden [6], [7]. Accumulating studies have demonstrated that vascular calcification is an active and biologically regulated process similar to osteogenesis, which is associated with the up-regulation of bone-associated proteins such as alkaline phosphatase (ALP), bone morphogenetic protein-2 (BMP-2), Matrix Gla protein (MGP) and muscle segment homeobox homolog (Msx2) [8]–[11].

A variety of osteogenic regulatory factors have been identified as being involved in the process of vascular calcification [8], [12]. Oxidized low density lipoprotein (Ox-LDL) is one of the important factors involved in vascular calcification [13]–[15]. Ox-LDL has been demonstrated to play a crucial role in the progression of atherosclerosis, and promote osteogenic differentiation and calcification of vascular smooth muscle cells (VSMCs) [13], [16]. Additionally, it has been shown that Ox-LDL stimulates the activation of neutral sphingomyelinase (N-SMase) which induces sphingomyelin hydrolysis and ceramide generation *in vitro* [17], [18]. Ceramide is an important signaling molecule involved in the regulation of cell differentiation and notably apoptosis [19]. Moreover, it is well known that Ox-LDL induces VSMC apoptosis [20]–[22], which is an important mechanism underlying vascular calcification [23]–[25]. Furthermore, high levels of ceramide have been identified in atherosclerotic plaques [26], [27]. Sphingomyelinase, also detected in atherosclerotic lesions,promotes atherosclerosis through hydrolysis of the sphingomyelin of LDL [28]. However, the role of N-SMase/ceramide in the progression of Ox-LDL-induced vascular calcification has not yet been established. Therefore, the aim of this study is to determine whether N-SMase/ceramide plays an important role in Ox-LDL-induced calcification of VSMCs.

## Materials and Methods

### Cell Culture

All cell culture reagents were purchased from Sigma Company, USA. Written informed consent was obtained from patients and approval was received from the Ethics Committee of the First Affiliated Hospital, Sun Yat-Sen University, China. VSMCs were isolated from human femoral arteries using the explant method as previously described [29]. VSMCs were maintained in Dulbecco's Modification of Eagle's Medium (DMEM) supplemented with 10% fetal bovine serum (FBS) at 37°C in 5% CO_2_. Cells between passages 3 and 6 were used in this study. To induce calcification, growth medium was replaced with osteogenic medium (DMEM supplemented with 10 mM beta-glycerophosphate).

### Low Density Lipoprotein Isolation and Oxidation

LDL was prepared using standard procedures as described [13]. Briefly, LDL (density 1.019–1.063 g/ml) was separated from human plasma by sequential density gradient ultracentrifugation. LDL fraction was dialyzed against PBS (pH 7.4) at 4°C for 24 hours. Oxidized LDL was prepared by incubation of LDL with 5 µM CuSO_4_ at 37°C for 3 hours and sterilized by passage through a 0.22 µm Millipore filter. Native LDL or Ox-LDL was used to treat cells for the indicated time periods.

### Mineralization Assay

Mineral deposition in cultured VSMCs was assessed by alizarin red staining. Calcified VSMCs were fixed in 4% formaldehyde for 10 minutes and exposed to 2% alizarin red (pH4.2) for 5 minutes at room temperature. Cells were washed with deionized water to remove excess dye and images were taken using an inverted phase contrast microscope. For alizarin red staining quantification, 10% formic acid was used to elute alizarin red dye and the absorbance at 405 nm was determined with a microplate reader and normalized to protein content. For quantification of calcium content, cells were washed with PBS and decalcified with 0.6 N HCl for 24 hours. Calcium content of the cultures was determined colorimetrically using the Ocresolphthalein complexone method as previously described [30] and normalized to protein content. The protein content was determined using BCA protein assay (Pierce, USA).

### Alkaline Phosphatase Activity Assay

VSMCs were seeded in 6-well plates at a density of 1×10^5^ cells/cm^2^ and cultured in osteogenic medium supplemented with or without Ox-LDL. Cells were harvested by three cycles of freeze-thaw in 0.1% Triton X-100 in PBS at indicated time points in presence of protease inhibitor cocktail (Sigma, USA). BCA™ protein assay (Pierce, USA) was used to quantify cellular protein concentration. Protein lysates (20 µg) were added to 180 µl p-NPP substrate and incubated for 15 minutes at 37°C. ALP activity was measured at 405 nm and calculated as nmol/ml p-nitrophenol converted per microgram of protein per minute.

### Determination of Neutral Sphingomyelinase Activity and Ceramide Level

N-SMAse activity was determined using [^14^C] sphingomyelin (Amersham, USA) as substrate as previously described [31]. Cells were harvested and homogenized by sonication at required time points. 100 µl of cell homogenate and 100 µl of substrate [^14^C] sphingomyelin were mixed in 0.1% Triton X-100, 20 mM HEPES buffer and incubated for 2 hours at 37°C. The released radioactive phosphocholine was quantified by liquid scintillation counting. For determination of ceramide level, the diacylglycerol (DAG) kinase (Calbiochem, USA) assay was performed as described [32]. Briefly, 10 µl of sample was added to assay buffer containing DAG kinase. 2 µCi of [γ-32p] ATP was then used to label the sample mixture for 30 minutes at room temperature. Phosphorylated lipids were extracted by the addition of chloroform: water (1∶1). Ceramide-1-phosphate and diacylglycerol-1-phosphate were thereafter separated using thin-layer chromatography and radioactive ceramide-1-phosphate was counted by liquid scintillation.

### Quantitative Real-time PCR

Total RNA was isolated from the cultured VSMCs using TRIzol Reagent (Invitrogen, USA), followed by reverse transcription using AMV Reverse Transcriptase (Roche, Germany). Quantitative PCR was performed using SYBR Green PCR Master Mix (Applied Biosystems, USA) in a StepOne Real Time PCR system (Applied Biosystems, USA). Primers used for quantitative PCR were as follows: β-actin (forward): CCAGCTCACCATGGATGATG, β-actin (reverse): GAGCCGTTGTCGACGACG; Msx2 (forward): TGGATGCAGGAACCCGG, Msx2 (reverse): AGGGCTCATATGTCTTGGCG; Osterix (forward): TAATGGGCTCCTTTCACCTG, Osterix (reverse): CACTGGGCAGACAGTCAGAA. Values were normalized to β-actin. The results were calculated using the comparative Ct (threshold cycle) method [33].

### Western Blot Analysis

Total protein was extracted from VSMC cultures and protein concentration was measured using a BCA protein assay kit (Pierce, USA). Equal amounts of protein were loaded and separated by 10% SDS-PAGE followed by transfer to a nitrocellulose membrane (Bio-Rad, USA). Membranes were blocked with 5% non-fat dried milk, followed by incubation with primary antibodies including phosphorylated Bcl2 (p-Bcl2), phosphorylated Bad (p-Bad) antibodies (Santa Cruz, USA), p38 MAPK or phosphorylated p38 MAPK antibodies (Cell signaling, USA) overnight at 4°C. Membranes were then washed and incubated with secondary antibodies conjugated to Horseradish Peroxidase (Dako, Denmark). Signals were detected by SuperSignal West Pico Chemiluminescent Substrate (Pierce, USA).

### Assessment of Cell Apoptosis

Apoptotic cells were counted by flow cytometry after cells were stained with Annexin V-FITC and propedium iodide (PI) using Annexin-V-FITC apoptosis detection kit (Sigma, USA) according to the manufacturer's instructions. Cells were harvested and washed twice with PBS. Cells were then resuspended in 1 × binding buffer and stained with Annexin V-FITC and PI for 10 minutes in the dark at room temperature. The fluorescence of the cells immediately was determined with a flow cytometer. Caspase-3 activity was quantified using fluorometric immunosorbent enzyme assay system as described in the manufacturer's instructions (Roche). Briefly, VSMCs were harvested in lysis buffer (1 × DTT) at required time points, and cell lysates were centrifuged to remove cellular debris. 100 µl aliquots of cell lysates were then added to fluorescence caspase substrate and the mixture samples were incubated for 1 hour at 37°C. The generated fluorochrome by proteolytic cleavage of the caspase substrate was measured by a plate reader with excitation at 355 nm and emission at 527 nm.

### Statistical Analysis

All results are expressed as mean ± SD. Statistical comparisons were made by one way ANOVA. P<0.05 was considered statistically significant.

## Results

### Ox-LDL Promotes VSMC Calcification in a Dose-dependent Manner

**Figure 1:**
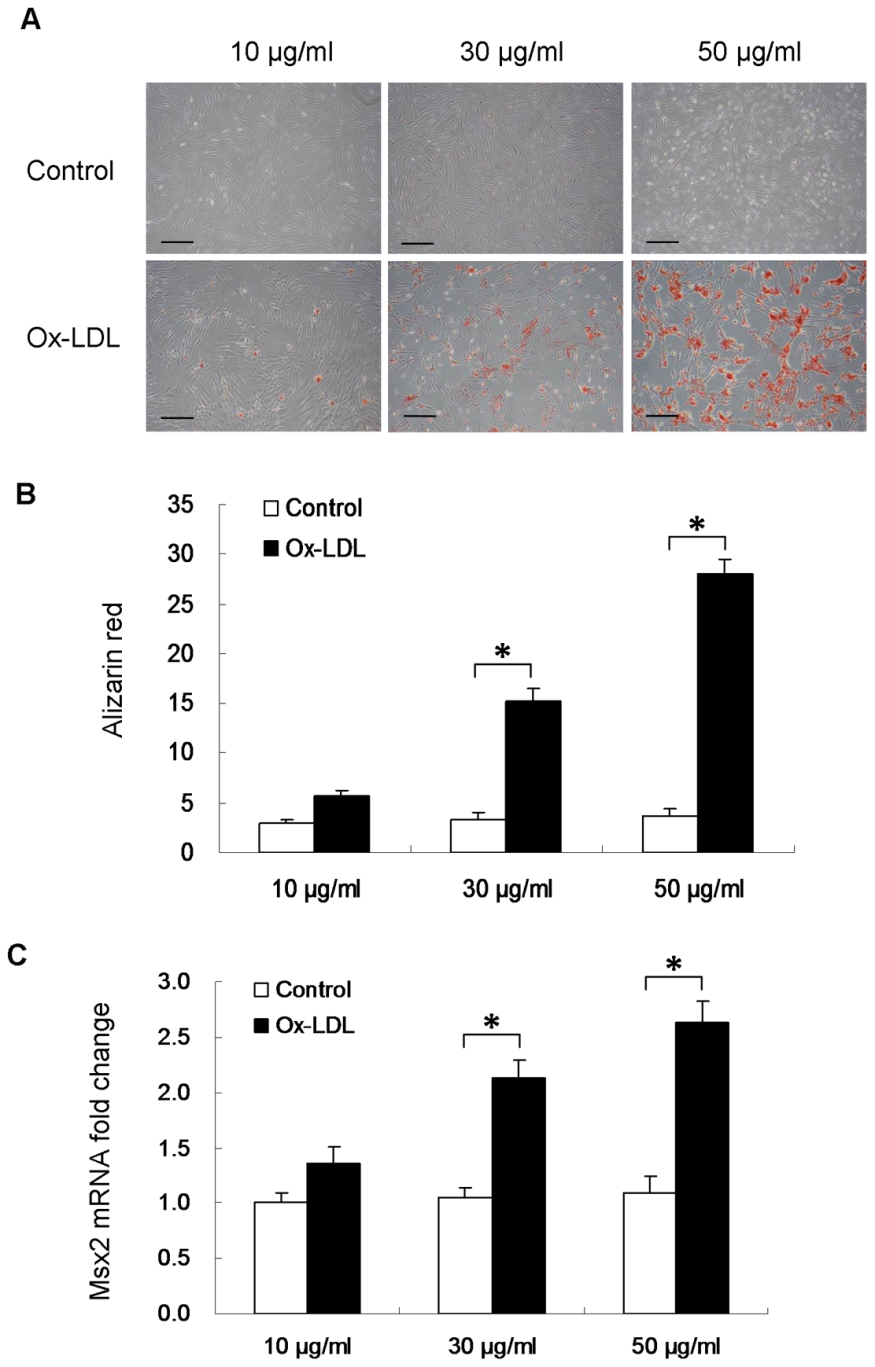
Dose-response analysis of the effect of Ox-LDL treatment on human VSMC calcification. Confluent human VSMCs were cultured in osteogenic medium in the presence of native LDL (control) or Ox-LDL (10 µg/ml, 30 µg/ml, 50 µg/ml) for 14 days (n = 3). (**A**) VSMCs were stained with alizarin red to assess mineralization. (**B**) Mineralization was quantified and shown as optical density units and normalized to protein content. (**C**) Msx2 mRNA levels were determined by quantitative real-time PCR. *P<0.001. Scale bar = 200 µm.

To determine the effect of Ox-LDL on vascular calcification, human VSMCs were treated with 10, 30, or 50 µg/ml Ox-LDL. Alizarin red staining was used to assess mineralization. As showed in Fig. 1A and 1B, Ox-LDL increased human VSMC calcification in a dose-dependent manner. However, no calcification was detected in native LDL-treated cells). Additionally, quantitative real-time PCR showed that Ox-LDL increased the osteogenic transcription factor, Msx2 mRNA expression in VSMCs by 2.1-fold at 30 µg/ml Ox-LDL and by 2.6-fold at 50 µg/ml Ox-LDL, respectively (Fig. 1C).

### Ox-LDL Increases Ceramide Levels in Cultured Human VSMCs

**Figure 2:**
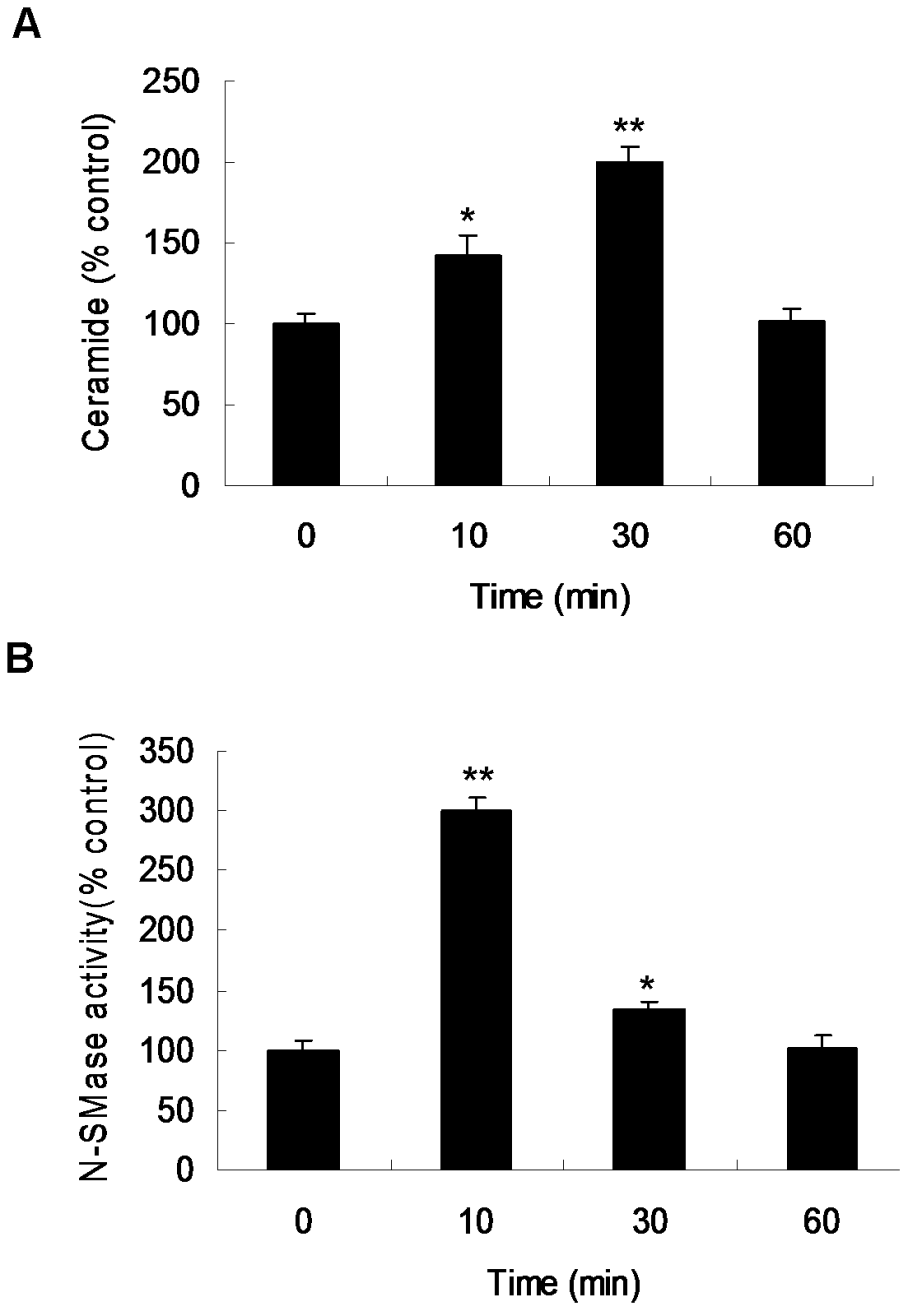
Effect of Ox-LDL on ceramide level and neutral sphingomyelinase (N-SMase) activity in VSMCs. Cells were incubated with 50 µg/ml Ox-LDL and harvested at indicated time points (n = 3). (**A**) Ceramide levels were determined using diacylglycerol kinase assay. (**B**) The activity of N-SMase was measured using ^14^C sphingomyelin as substrate. *p<0.01, **p<0.001 vs. control.

To investigate the effect of Ox-LDL on ceramide levels, 50 µg/ml Ox-LDL was used to treat VSMCs. Ox-LDL treatment for 10 minutes increased the level of ceramide in cultured VSMCs by 1.4-fold and reached maximal level of ceramide (2-fold) by 30 minutes, compared with control cells (Fig. 2A). In addition, a 3-fold increase in N-SMase activity in VSMCs was detected after Ox-LDL treatment for 10 minutes, and there was a 1.3-fold increase in N-SMase activity at 30 minutes (Fig. 2B).

### N-SMase Inhibitor, GW4869, Attenuates Ox-LDL-induced VSMC Calcification

**Figure 3:**
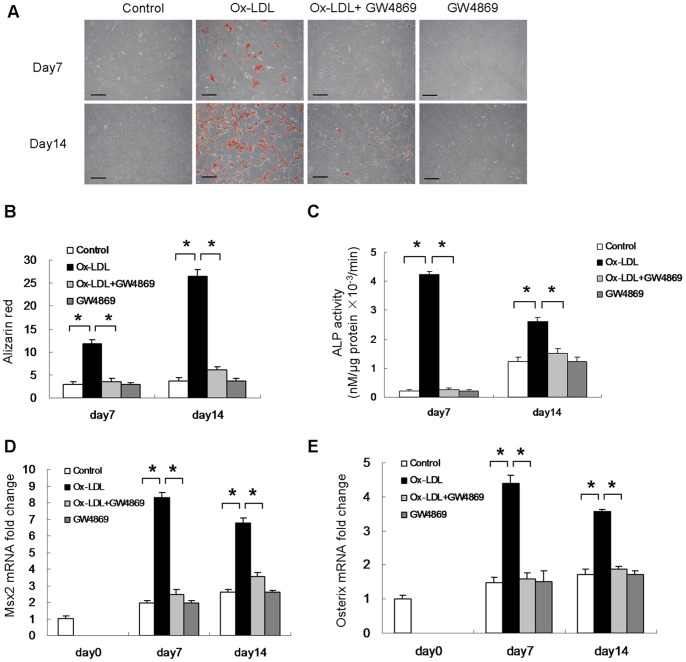
Effect of GW4869 on Ox-LDL-induced VSMC calcification. Cells were treated with 50 µg/ml Ox-LDL (in DMSO) for 14 days. N-SMase inhibitor, GW4869 (20 µM in DMSO) was used to treat cells in the presence of Ox-LDL (n = 3). (**A**) Alizarin red was used to assess mineralization (bar = 200 µm). (**B**) Quantification of mineral deposition was performed. (**C**) ALP activity was measured by spectrophotometry. (**D&E**) Msx2 and Osterix mRNA levels were determined by quantitative real-time PCR. *p<0.01.

Since Ox-LDL stimulates N-SMase activity and increases the level of ceramide in cultured VSMCs, we decided to investigate whether N-SMase/ceramide signaling is involved in Ox-LDL-induced mineralization of VSMCs. GW4869, a specific inhibitor of N-SMase, was used to treat VSMCs in the presence of Ox-LDL. Alizarin red staining showed that inhibition of N-SMase attenuated Ox-LDL-induced mineralization of VSMCs (Fig. 3A). Quantification of alizarin red staining showed that Ox-LDL increased mineralization by 3.9-fold at day7, and by 7.1-fold at day14, respectively. However, addition of GW4869 caused a reduction of mineralization by 71% at day7, and by 77% at day14, respectively (Fig. 3B). Similarly, ALP activity, which is an early osteogenic differentiation marker, was also increased by 20-fold and 2.1-fold in Ox-LDL-treated cells at day7 and day14, respectively. However, reduced levels of ALP activity were detected in cells treated with GW4869, compared with control cells (Fig. 3C). In addition, we found that Ox-LDL increased Msx2 and Osterix mRNA expression in VSMCs. GW4869 treatment of VSMCs significantly down-regulated Msx2 and Osterix mRNA expression, compared with cells treated with Ox-LDL alone (Fig. 3D & E).

### Ceramide Treatment Stimulates VSMC Calcification

**Figure 4:**
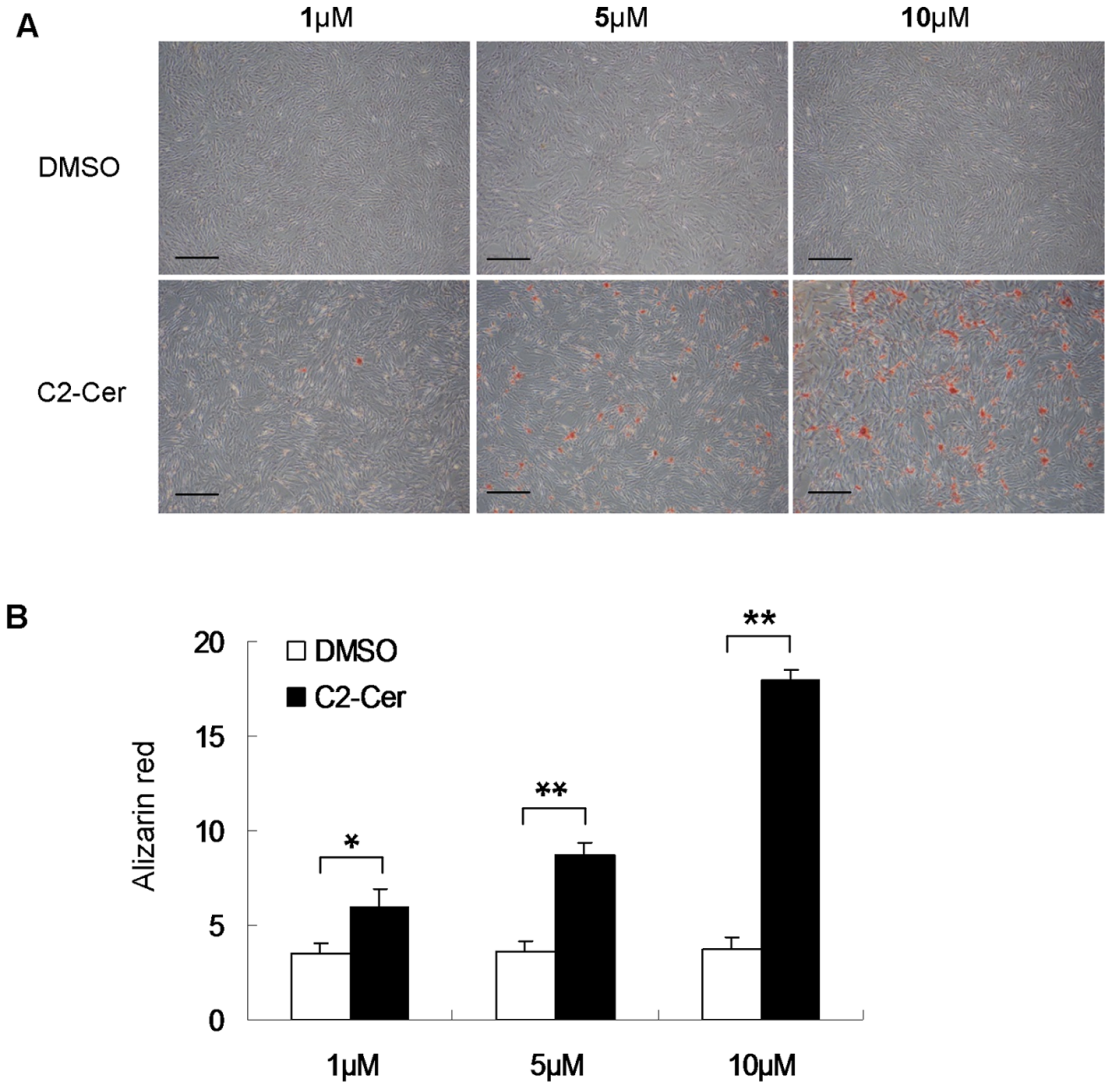
Effect of C2-ceramide on VSMC calcification. Human VSMCs were incubated in the presence of 1 µM, 5 µM, 10 µM of C2-ceramide (C2-Cer) for 14 days (n = 3). (**A**) Alizarin red was used to visualize VSMC mineralization (bar = 200 µm). (**B**) Quantification of mineral deposition was performed. *p<0.05. **p<0.001.

Next, we investigated effect of C2-ceramide on VSMC calcification. C2-ceramide was used to treat VSMCs under osteogenic condition for 14 days. Alizarin red staining showed that C2-ceramide accelerated calcification of human VSMCs in a dose-dependent manner (Fig. 4A). Quantification of alizarin red staining showed C2-ceramide increased mineralization in VSMCs by 1.7-fold, 2.4-fold and 4.8-fold at a concentration of 1 µM, 5 µM and 10 µM, respectively (Fig. 4B).

### Ceramide Treatment Accelerates Ox-LDL-induced VSMC Calcification

**Figure 5:**
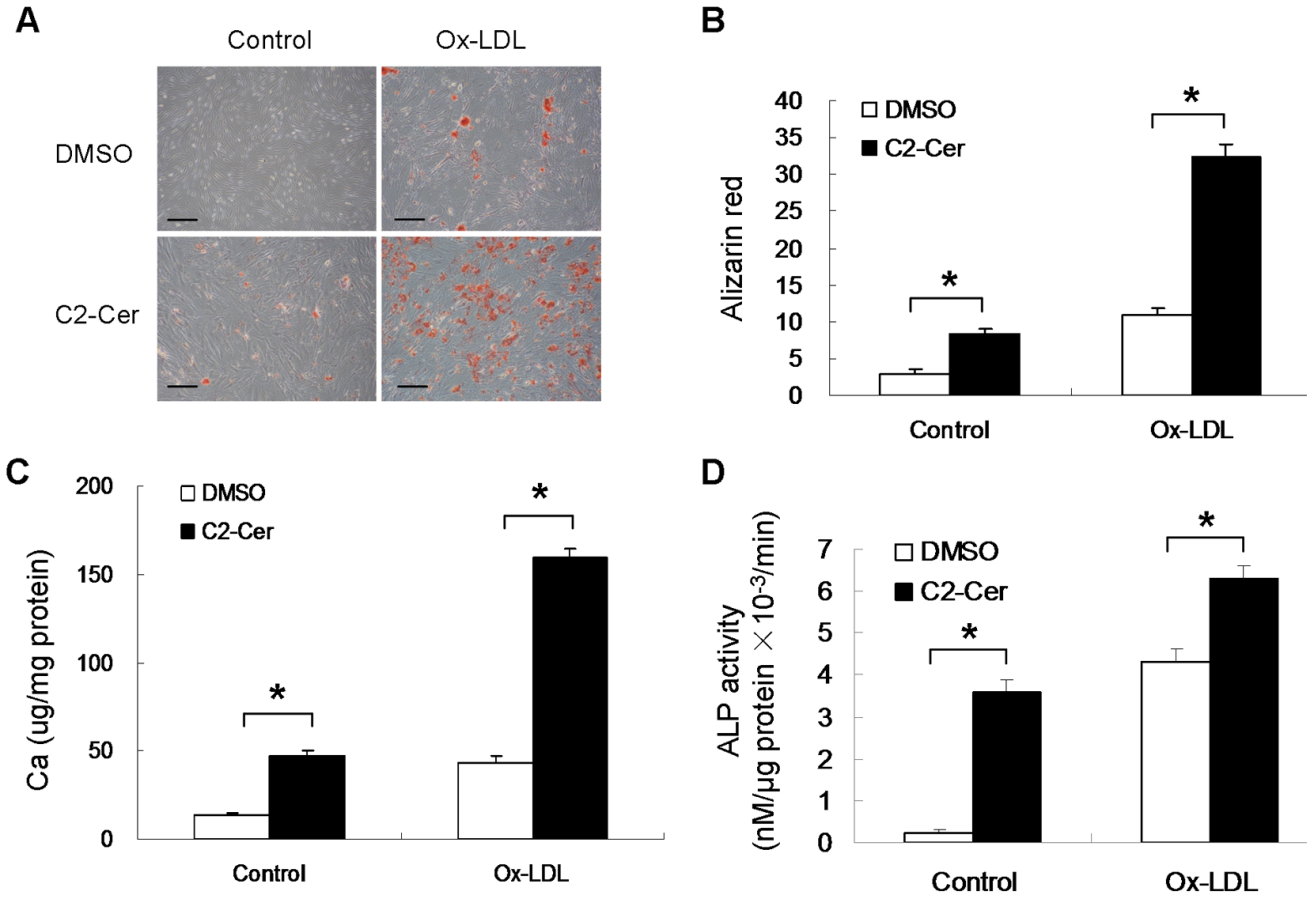
Ox-LDL-induced VSMC calcification was enhanced by ceramide treatment. Cells were treated with 50 µg/ml Ox-LDL alone or 50 µg/ml Ox-LDL in the presence of 5 µM C2-ceramide for 7 days (n = 3). (**A**) Cells were stained with alizarin red (bar = 200 µm). (**B**) Quantification of mineral deposition was performed. (**C**) The calcium content was measured using ocresolphthalein complexone method. (**D**) ALP activity was measured by spectrophotometry. *p<0.01.

To further confirm that N-SMase/ceramide signaling is involved in Ox-LDL-induced vascular calcification, C2-ceramide was used to treat VSMCs in the presence of Ox-LDL. Cells treated with C2-ceramide in the presence of Ox-LDL showed a 3-fold increased mineralization at day7, compared with cells treated with Ox-LDL alone (Fig. 5A and 5B). As shown in Fig. 5C, Ox-LDL-induced calcium deposition was enhanced 3.2-fold by C2-ceramide treatment. Furthermore, ALP activity was increased by 1.5-fold at day7 in cell treated with C2-ceramide together with Ox-LDL, compared with cells treated with Ox-LDL alone (Fig. 5D).

### Ox-LDL-induced Apoptosis is Ceramide-dependent

**Figure 6:**
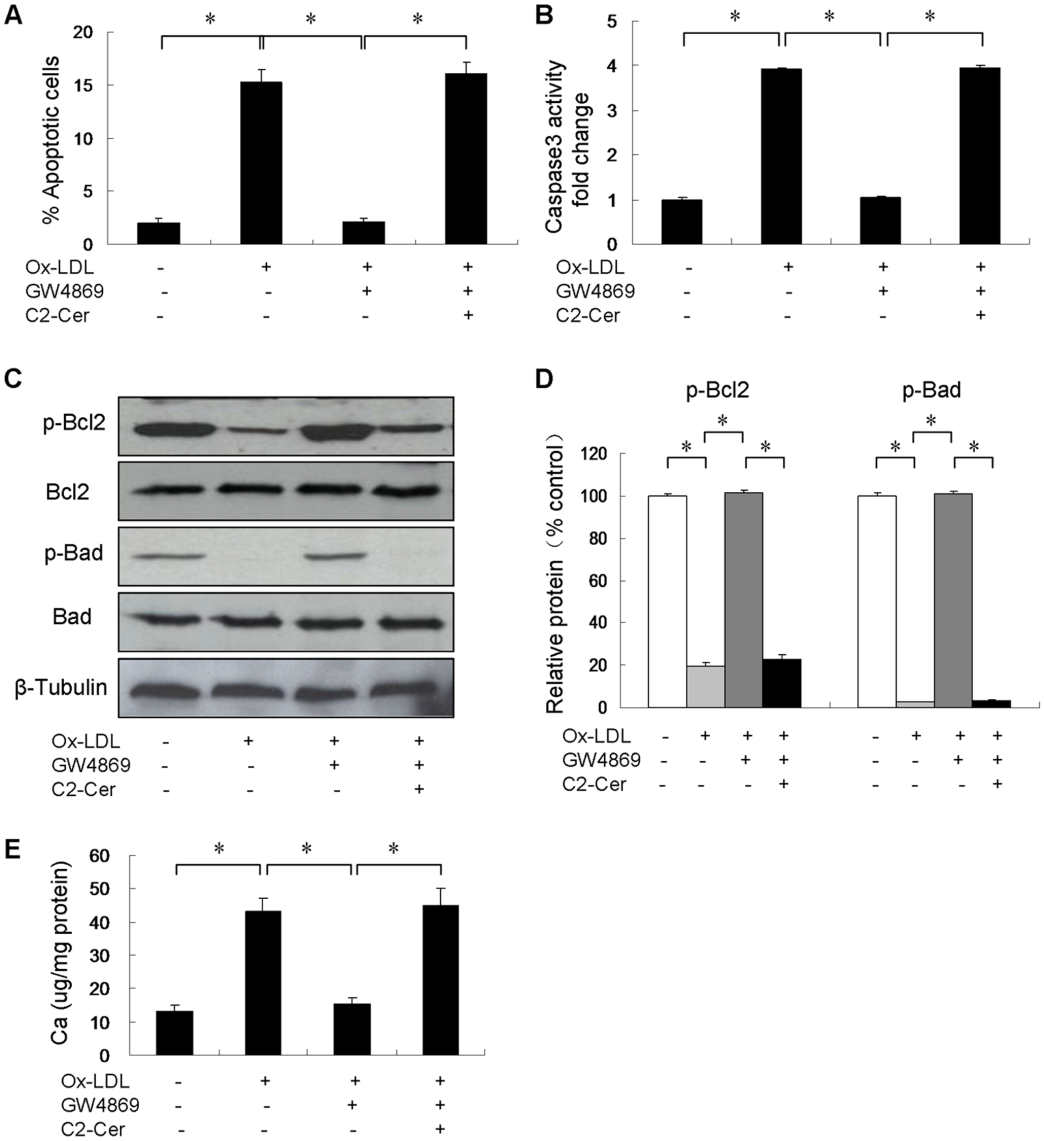
Effect of GW4869 on Ox-LDL-induced apoptosis in cultured VSMCs (n = 3). Cells were incubated in control medium (in DMSO), 50 µg/ml Ox-LDL alone (in DMSO), 50 µg/ml Ox-LDL in the presence of 20 µM GW4869 (in DMSO) or 50 µg/ml Ox-LDL in the presence of 20 µM GW4869 and 5 µM C2-ceramide (in DMSO) for 24 hours. (**A**) The percentage of apoptotic cells was then assessed. (**B**) Caspase-3 activity was measured and results are shown as percentage of control. (**C**) p-Bcl2 and p-Bad protein levels were detected by immunoblot analysis. (**D**) p-Bcl2 and p-Bad protein levels were quantified and normalized to total Bcl2 and Bad levels. (**E**) Cells were treated with/without 20 µM GW4869 and 5 µM C2-ceramide in the presence of Ox-LDL for 7 days. The calcium content was quantified using ocresolphthalein complexone method. *p<0.01.

Since Ox-LDL has been shown to stimulate both ceramide generation and apoptosis, we decided to investigate whether apoptosis is required for Ox-LDL-induced VSMC mineralization. Ox-LDL treatment showed an increased rate of VSMC apoptosis. Addition of N-SMase inhibitor, GW4869 significantly reduced apoptosis in the presence of Ox-LDL, an effect which was bypassed by C2-ceramide (Fig. 6A). Similarly, Ox-LDL treatment of cells increased caspase-3 activity by 3.9-fold, an effect which was prevented by GW4869 (Fig. 6B). Next, Western blot analysis showed that Ox-LDL reduced anti-apoptotic phosphorylated Bcl2 and Bad expression. However, GW4869 stimulated Bcl2 and Bad phosphorylation, and this effect was reversed by C2-ceramide (Fig. 6C and D). Moreover, GW4869 significantly reduced Ox-LDL-induced calcium deposit, an effect which was reversed by C2-ceramide (Fig. 6E).

### Apoptosis is Required for Ox-LDL-induced VSMC Calcification

**Figure 7:**
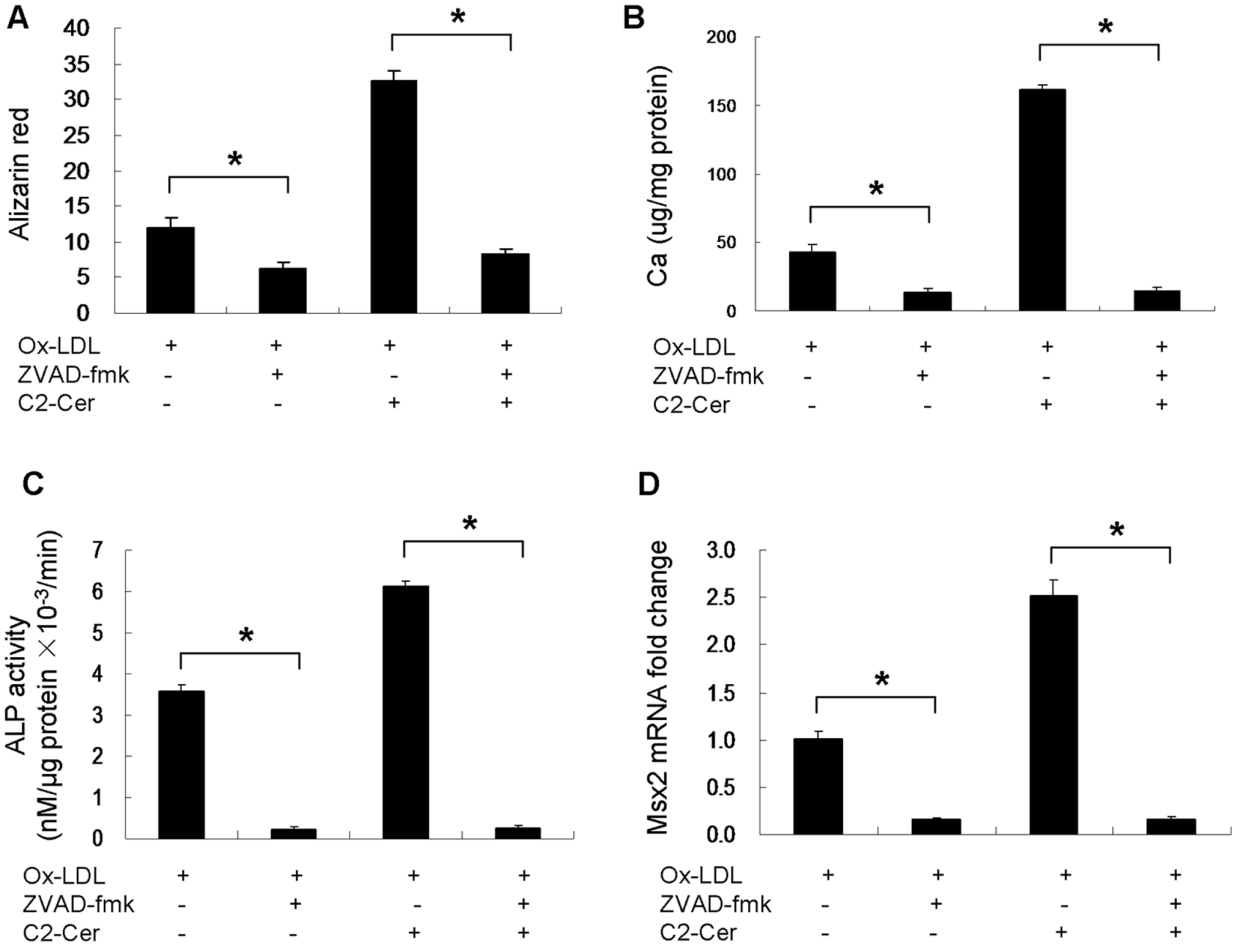
Effect of ZVAD-fmk on Ox-LDL-induced mineralization in cultured VSMCs (n = 3). Cells were treated with 5 µM ZVAD-fmk (a caspase inhibitor) in the presence of Ox-LDL for 7 days. (**A**) Cells were stained with alizarin red and quantification of mineral deposition was performed at indicated times. (**B**) The calcium content was measured using ocresolphthalein complexone method. (**C**) ALP activity was measured by spectrophotometry. (**D**) Msx2 mRNA levels were determined by quantitative real-time PCR after cells were treated with/without ZVAD-fmk. *p<0.01.

To determine whether apoptosis is involved in Ox-LDL-induced calcification, caspase inhibitor, ZVAD-fmk was used to treat cells in the presence of Ox-LDL. Quantification of alizarin red staining showed a 50% reduction in mineralization after ZVAD-fmk treatment for 7 days in the presence of Ox-LDL and a 74% reduction in mineralization in the presence of C2-ceramide and Ox-LDL (Fig. 7A). Similarly, calcium quantification showed that ZVAD-fmk reduced calcium deposit by 68% in the presence of Ox-LDL and 91% in the presence of C2-ceramide and Ox-LDL (Fig. 7B). Additionally, ALP activity was significantly reduced by ZVAD-fmk under both Ox-LDL condition and Ox-LDL together with C2-ceramide condition (Fig. 7C). Furthermore, ZVAD-fmk addition caused an 84% and 93% reduction of Msx2 mRNA expression in the presence of Ox-LDL, and in the presence of C2-ceramide and Ox-LDL, respectively (Fig. 7D).

### Activation of p38 MAPK Signaling is Critical for Ox-LDL-induced VSMC Calcification

**Figure 8:**
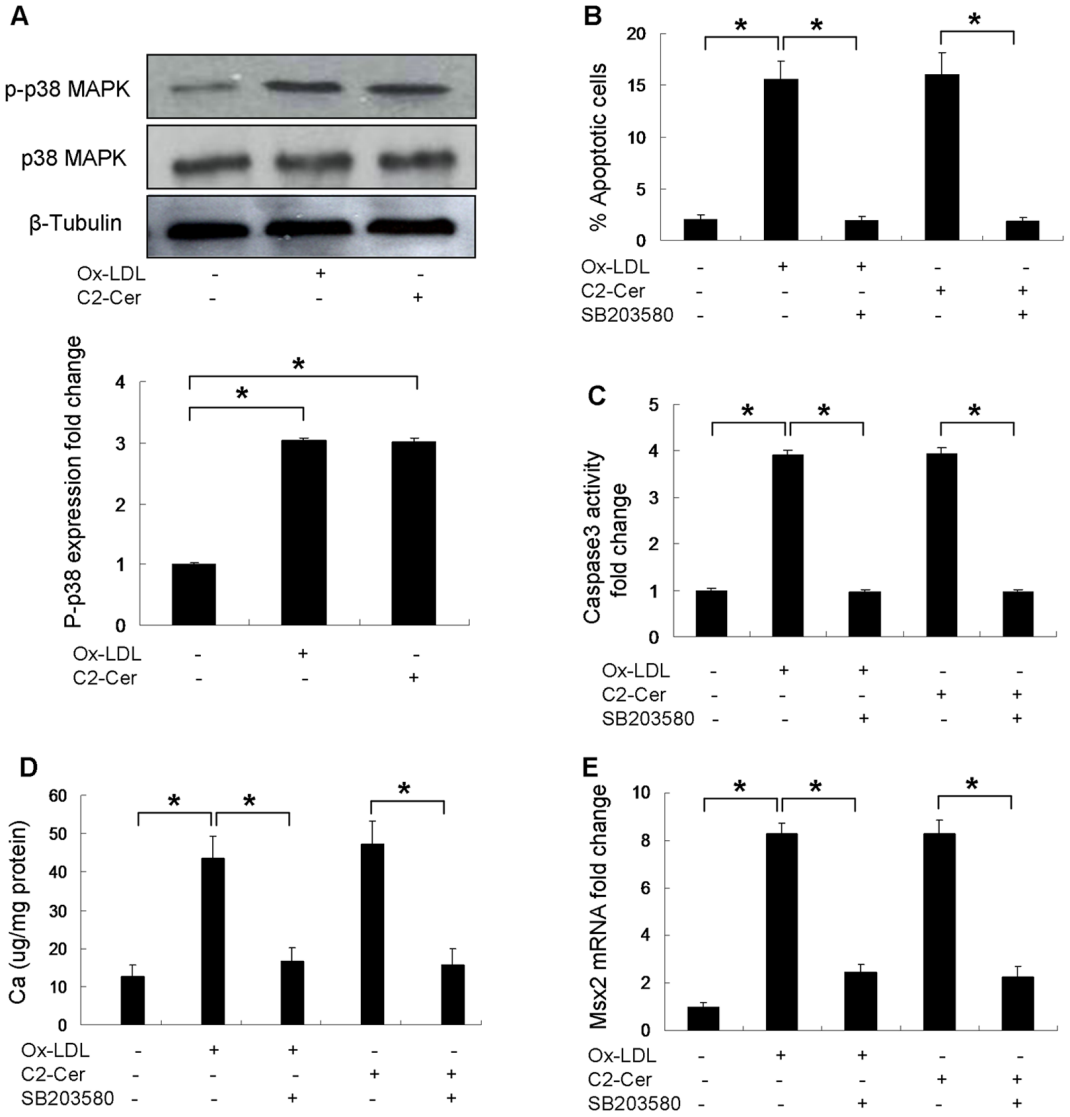
Effect SB203580 of on Ox-LDL-induced mineralization in cultured VSMCs (n = 3). Cells were grown in control medium (in DMSO), 50 µg/ml Ox-LDL alone (in DMSO), 50 µg/ml Ox-LDL supplemented with 5 µM SB203580 (in DMSO), 5 µM C2-ceramide alone (in DMSO) or 5 µM C2-ceramide supplemented with 5 µM SB203580 (in DMSO) for 24 hours. (**A**) p-p38 MAPK protein levels were detected by immunoblot analysis. (**B**) The percentage of apoptotic cells was then assessed. (**C**) Caspase-3 activity was measured in a parallel experiment. Cells were treated with SB203580 in the presence of Ox-LDL for 7 days. (**D**) The calcium content was measured using ocresolphthalein complexone method. (**E**) Msx2 mRNA expression was determined by quantitative real-time PCR after cells were treated with/without SB203580. *p<0.01.

Since p38 mitogen-activated protein kinase (MAPK) plays a very important role in apoptotic cell death, we further investigated whether p38 MAPK signaling is required for Ox-LDL-induced VSMC calcification. SB203580, a specific inhibitor of p38 MAPK was used to treat cells in the presence of Ox-LDL. Both Ox-LDL and C2-ceramide treatment increased the phosphorylation of p38 MAPK (Fig. 8A). Addition of SB203580 significantly reduced apoptosis in the presence of Ox-LDL or C2-ceramide (Fig. 8B). Similarly, SB203580 treatment of cells decreased caspase-3 activity induced by Ox-LDL or C2-ceramide (Fig. 8C). Calcium quantification showed that inhibition of p38 MAPK by SB203580 attenuated calcium deposit in the presence of Ox-LDL or C2-ceramide (Fig. 8D). Additionally, SB203580 addition caused a decreased Msx2 mRNA expression in the presence of Ox-LDL or C2-ceramide (Fig. 8E).

## Discussion

**Figure 9:**
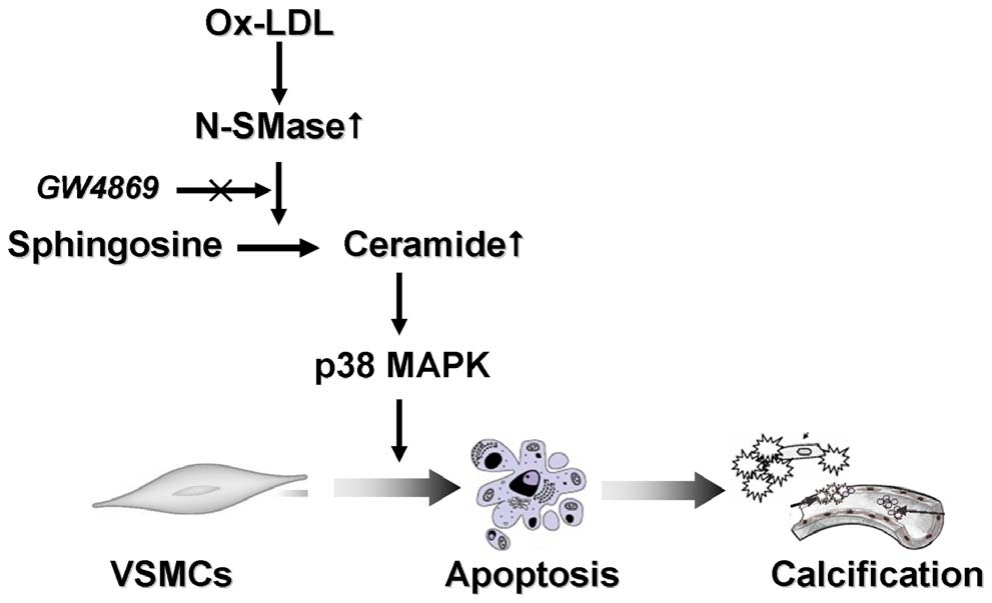
Proposed model for the regulatory interplay of Ox-LDL, N-SMase, ceramide and p38 MAPK during the process of VSMC calcification. Ox-LDL stimulates the activity of N-SMase, which results in increased levels of ceramide. As a result, the activated ceramide signaling stimulates p38 MAPK and triggers VSMC apoptosis, leading to VSMC calcification, which can be attenuated by GW4869 treatment.

In this present study, we for the first time report that ceramide accelerates vascular calcification and N-SMase/ceramide signaling plays an important role in Ox-LDL-induced VSMC calcification via p38 MAPK signaling (Figure 9). We show that Ox-LDL promotes osteogenic differentiation and calcification of VSMCs and up-regulates Msx2 expression, which is a key factor of vascular calcification [34], [35]. These findings are consistent with previous studies [13], [14], [16]. Inhibition of N-SMase activity strongly reduced Ox-LDL-induced apoptosis and calcification of VSMCs. Ceramide treatment accelerated Ox-LDL-induced VSMC mineralization. Furthermore, inhibition of apoptosis significantly attenuated Ox-LDL and C2-ceramide induced mineralization. Both Ox-LDL and ceramide treatment stimulated phosphorylation of p38 MAPK. Inhibition of p38 MAPK reduced apoptosis and attenuated Ox-LDL- and ceramide-induced calcification in VSMCs.

N-SMase, which hydrolyzes sphingomyelin to generate ceramide, is involved in the induction of ceramide-mediated proapoptotic signaling under a variety of conditions [36]. A previous study has demonstrated that usage of GW4869, an N-SMase inhibitor, completely inhibits VSMC apoptosis and ceramide generation induced by a component of lipoprotein, apolipoprotein C-I [37]. In addition, usage of N-SMase antibody abrogated the Ox-LDL-induced apoptosis. Increased activity of N-SMase was also correlated with elevated levels of ceramide and apoptosis in plaque and calcified plaques in patients. Ox-LDL-induced apoptosis in VSMCs involves the activation of N-SMase, and ceramide generation [38]. Similarly, we found that GW4869 inhibits Ox-LDL-induced apoptosis in VSMCs, and this effect was prevented by C2-ceramide, suggesting that Ox-LDL activates N-SMase to generate ceramide, and induces apoptosis. Furthermore, the finding that inhibition of N-SMase activity by GW4869 significantly reduced Ox-LDL-induced calcification of VSMCs and prevented the up-regulation of Msx2 expression induced by Ox-LDL supports our hypothesis that N-SMase is involved in Ox-LDL-induced mineralization of VSMCs.

The sphingolipid ceramide is an important second signal molecule involved in cell differentiation and apoptosis [19], [39]. Ceramide plays a key role as a mediator in Ox-LDL-induced apoptotic signaling pathway. Since ceramide can stimulate apoptosis, which initiates vascular calcification. It is tempting to speculate that the procalcific effect of Ox-LDL is mediated by the stimulation of ceramide-activated apoptosis. To determine whether ceramide plays a critical role in vascular calcification, we detected the effect of Ox-LDL on ceramide generation *in vitro*. Interestingly, ceramide level is significantly increased after Ox-LDL treatment for 10 minutes and reaches maximal level by 30 minutes. Next, we used C2-ceramide to treat VSMCs in the presence of Ox-LDL for 7 days. Stronger mineralization was detected in cells treated with C2-ceramide and Ox-LDL at day7, whereas little mineralization was detected in cells treated with Ox-LDL alone. Apparently, Ox-LDL-induced mineralization of VSMCs is markedly enhanced by concomitant treatment with C2-ceramide. Ox-LDL-induced ALP activity is also further enhanced by C2-ceramide treatment. These findings suggest that ceramide is involved in Ox-LDL-induced VSMC calcification.

The present observation that Ox-LDL induces apoptosis of VSMCs is consistent with the results by Bachem et al [40], but it is different from the finding by Auge et al. that Ox-LDL promotes proliferation of VSMCs [17]. Ox-LDL may lead to proliferation or apoptosis of VSMCs depending on concentration and the extent of oxidation, and cell density. High concentration of Ox-LDL induces apoptosis of VSMCs [40], whereas low concentration of Ox-LDL may promote proliferation of VSMCs. A previous study has demonstrated that VSMC apoptosis accelerates plaque growth in atherosclerosis and promotes vascular calcification [41]. *In vitro* studies have also shown that apoptotic VSMCs are associated with vascular calcification [23], [42]. In this study, we show that Ox-LDL stimulates VSMC apoptosis by inhibiting the phosphorylation of Bcl2 and Bad, which is prevented by N-SMase inhibitor, GW4869. In contrast, the proapoptotic effect of Ox-LDL is not prevented by GW4869 in the presence of C2-ceramide, suggesting that Ox-LDL-induced apoptosis is ceramide-dependent and Ox-LDL activates VSMC apoptosis through N-SMase/ceramide signaling. Furthermore, inhibition of apoptosis by caspase inhibitor, ZVAD-fmk, strongly attenuated Ox-LDL and C2-ceramide-induced mineralization, and reduced the expression of Msx2, suggesting that ceramide-mediated VSMC apoptosis is involved in Ox-LDL-induced VSMC calcification. Ceramide has been shown to increase p38 MAPK phosphorylation [43]. In this study, we also found that C2-ceramide stimulated p38 MAPK phosphorylation. Accumulating studies have shown that p38 MAPK plays a very important role in apoptotic cell death and vascular calcification [44]–[47]. Similarly, we found that inhibition of p38 MAPK by SB203580 prevented Ox-LDL- and C2-ceramide-induced apoptosis, and attenuated Ox-LDL- and C2-ceramide-induced calcification, suggesting that p38 MAPK is involved in Ox-LDL- and C2-ceramide-induced calcification of VSMCs.

In summary, we have shown that N-SMase/ceramide is involved in Ox-LDL-mediated vascular calcification via p38 MAPK signaling. Therefore, N-SMase/ceramide could act as a novel potential therapeutic target to treat vascular calcification with increased oxidative stress. Further studies are needed to elucidate the signaling pathway by which Ox-LDL activates N-SMase, generates ceramide, and induces apoptosis during vascular calcification.
